# Chimeric antigen receptor-modified T Cells inhibit the growth and metastases of established tissue factor-positive tumors in NOG mice

**DOI:** 10.18632/oncotarget.14367

**Published:** 2016-12-30

**Authors:** Qing Zhang, Haiyu Wang, Huizhong Li, Jinjing Xu, Kang Tian, Jie Yang, Zheng Lu, Junnian Zheng

**Affiliations:** ^1^ Cancer Institute, Xuzhou Medical University, Xuzhou, Jiangsu, 221002, China; ^2^ Jiangsu Center for the Collaboration and Innovation of Cancer Biotherapy, Cancer Institute, Xuzhou Medical College, Xuzhou, Jiangsu, 221002, China

**Keywords:** chimeric antigen receptor, T cell, tissue factor, lung cancer, melanoma

## Abstract

Chimeric antigen receptor (CAR)-modified T cell (CAR T) is a promising therapeutic option for patients with cancer. Such an approach requires the identification of tumor-specific antigen targets that are expressed in solid tumors. We developed a new third-generation CAR directed against tissue factor (TF), a surface molecule overexpressed in some types of lung cancer, melanoma and other cancers. First, we demonstrated by immunohistochemistry that TF was overexpressed in squamous cell carcinoma and adenocarcinoma of non-small cell lung cancer (NSCLC) and melanoma using a human tissue microarray. In the presence of TF-positive cancer cells, the CAR-modified T cells (TF-CAR T) were highly activated and showed specific cytotoxicity to TF-positive cancer cells *in vitro*. In established s.c. xenograft and lung metastasis models, TF-CAR T cells could significantly suppress the growth of s.c. xenograft and metastasis of TF-positive cancer cells. Additionally, the safety evaluation of TF-CAR T cells *in vivo* showed that the treatment did not cause obvious toxicity in mice. Taken together, these findings indicate that TF-CAR T cells might be a novel potential therapeutic agent for the treatment of patients with TF-positive cancers.

## INTRODUCTION

The use of chimeric antigen receptor (CAR) modified T cells (CAR T) for the treatment of cancers is an emerging field. The genetic modification of primary T cells with chimeric antigen receptors can redirect T cells against cancer cells [1]. The adoptive transfer of these cancer-specific T cells into patients provides a novel way to deliver specific antigen-targeted cancer therapy; this approach has been proven effective against B cell cancers in clinical trials [2, 3]. In the past two years, three CAR T cell therapies, namely, CTL019 (Novartis), JCAR015 (Juno Therapeutics) and KTE-C19 (Kite Pharma, Amgen), have been granted FDA breakthrough therapy status. However, the CAR T therapy approach for the treatment of solid tumors has not yet been granted this status. Such an approach requires the identification of tumor-specific antigen targets that are expressed in solid tumors. One such potential molecular target is tissue factor (TF).

TF, also named coagulation factor III, is overexpressed in many types of tumors [4–9]. The TF levels in clinical samples of numerous types of human cancers are up to 1000-fold greater than those of their normal counterparts, with only a few exceptions (e.g., renal cancer) [10]. Under normal conditions, TF is also expressed on extravascular cells of several normal tissues and in the adventitial layer of large blood vessel walls [11, 12]. However, TF is anatomically sequestered from its natural ligand coagulation factor VII (FVII) and from TF-targeting therapeutic agents circulating in blood by the intact semipermeable endothelial layer of normal blood vessels [13]. In contrast, because the walls of the tumor vasculature are leaky, systemically administered TF-targeting therapeutic agents can access TF on tumor cells via the leaky tumor neovasculature [13, 14]. Therefore, TF-targeting therapies could be used to eradicate tumor cells [13].

Anti-TF immunotherapeutic chimeric antibody and other therapeutics targeting TF have been reported previously [6, 15–20]. The TF-specific antibody-conjugated drug HuMax-TF-ADC for treating TF-positive solid tumors is in a phase I clinical trial (NCT02552121). Others and we have previously shown that the light chain of FVII retained the affinity of original FVII to its receptor TF [21, 22]. The light chains of the human and mouse FVII-conjugated drugs (hlFVII-LDM, mlFVII-LDM) that we developed showed effective growth suppression of TF-positive human lung cancer and mouse colon cancer, respectively [22, 23].

In the mouse model of human xenografts, the TF targets include human TF expressed by tumor cells and mouse TF expressed by other potential normal tissues. Because mouse FVII binds strongly to both human TF and mouse TF, unlike human FVII, which binds strongly to human TF but weakly to mouse TF [24], the light chain of mouse FVII (mlFVII) was chosen as the targeting domain of the CAR in this study. This mouse model can somewhat simulate the behavior of CAR T with hlFVII as the target vehicle in the human body.

In this study, we generated the first CAR against TF (TF-CAR), which consists of mlFVII, the hinge and transmembrane domains of human CD8α, and the intracellular signal domains of human CD28, 4-1BB and CD3ζ chain. Human primary T cells modified with the CAR (TF-CAR T) were then tested against non-small cell lung cancer (NSCLC) cells and melanoma cells *in vitro* and in xenograft and metastasis models of human NSCLC in NOG mice.

## RESULTS

### TF expression in human cancer tissues and cancer cell lines

**Figure 1:**
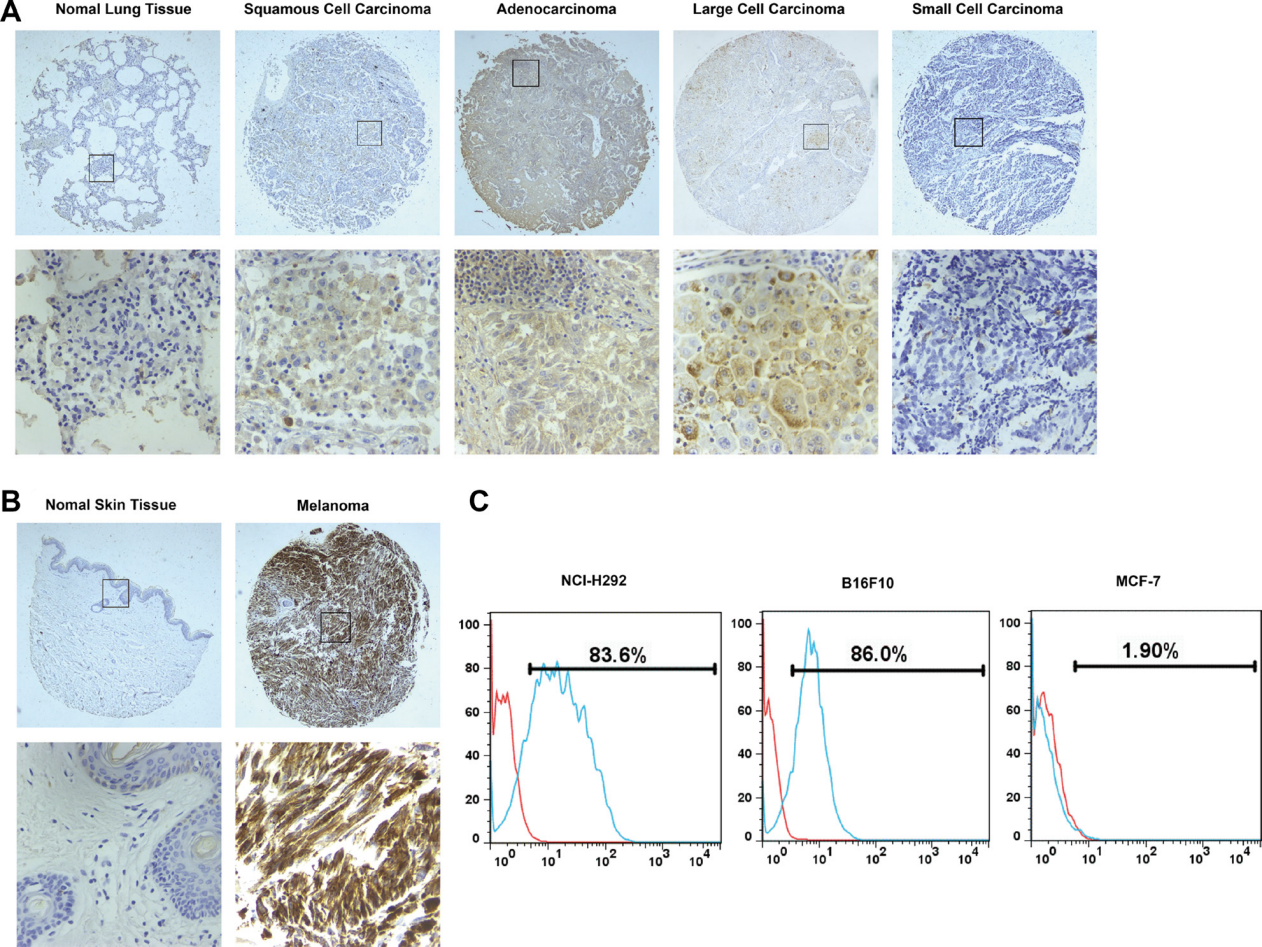
TF expression in human samples and cancer cell lines (**A**) and (**B**) Tissue microarray analysis of TF expression in human samples by IHC. Representative photographs were taken under different magnifications (Top panel, ×100; bottom panel, ×400). (**C**) Flow cytometry analysis of TF expression in cancer cell lines.

Because there have been conflicting reports about TF expression in lung cancer and melanoma [25–29], we examined TF expression in human tissue microarray slides of melanoma and lung cancer tissues and their corresponding normal tissues by immunohistochemistry (IHC). As shown in Table 1, TF expression in NSCLC, including squamous cell carcinoma and adenocarcinoma, was commonly higher than that in normal lung tissue. This difference was statistically significant (*p* = 0.008, *p* = 0.032, respectively). Interestingly, TF expression in small-cell lung cancer tissue was lower than that in normal lung tissue. Our results also showed a significantly higher level of TF expression in melanoma tissue than in corresponding normal skin tissues (*p* < 0.0001). Representative images are presented in Figure 1A.

**Table 1:** Levels of tissue factor in human samples

Tissue type	*n*	Intensity (mean)	Pos.cells (mean)	IRS (mean)	IRS ≤ 3	IRS > 3	*P*
Normal lung tissue	26	0.65	2.58	2.38	13	13	
Small cell carcinoma	49	0.33	0.18	1.08	39	10	0.011
Non-small cell lung cancer	49	1.67	2.39	5	28	21	0.049
Squamous cell carcinoma	39	1.74	2.31	4.97	22	17	0.008
Adenocarcinoma	10	1.4	2.7	5.1	6	4	0.032
Normal skin tissue	48	0.5	2.19	1.75	35	13	
Malignant melanoma	80	1.59	3.08	5.31	25	55	< 0.0001

*n*, number of samples; Intensity, staining intensity; Pos.cells, the number of positive cells; IRS, immunoreactive score. *P*-value was obtained by comparing with normal tissue.

Additionally, TF expression on the surface of cancer cells was determined. As shown in Figure 1B, TF is overexpressed on the surface of human NSCLC NCI-H292 cells and mouse melanoma B16F10 cells, while its expression on the surface of human breast cancer MCF-7 cells was negligible.

### Preparation of TF-CAR T cells

**Figure 2:**
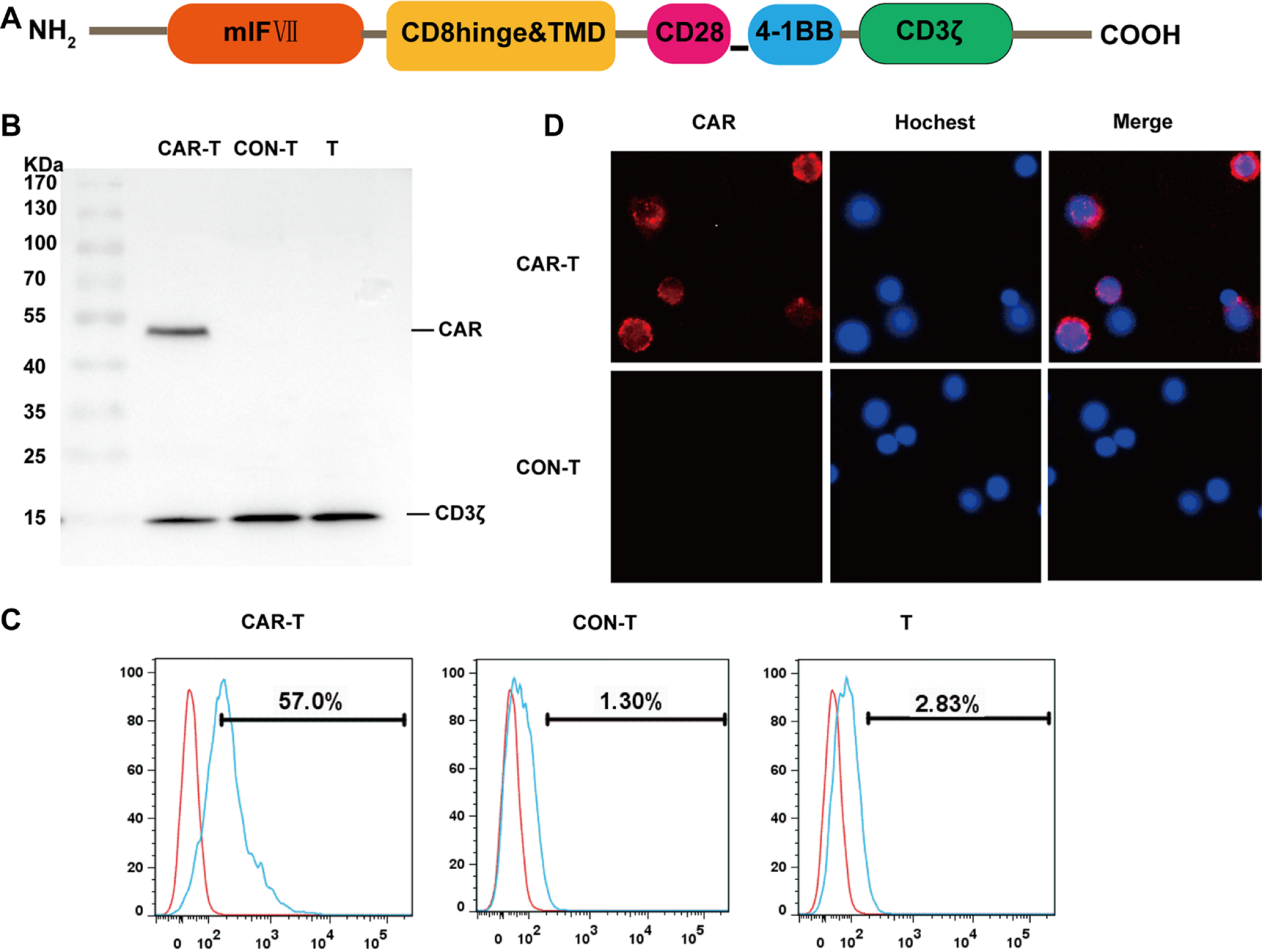
Generation and characterization of TF-CAR T cells *in vitro* (**A**) Structure diagram of TF-CAR. mlF VII, the light chain of mouse coagulation factor VII; TMD, transmembrane domain. (**B**) Western bloting analysis of TF-CAR expression in T cells. CAR-T, TF-CAR T cells; CON-T, vector-transduced T cells; T, untreated T cells. (**C**) Flow cytometry analysis of TF-CAR expression in transduced T cells. (**D**) Immunofluorescence staining analysis of TF-CAR expression in transduced T cells. The images were taken under × 400 magnification. (**E**) CAR positive ratio analysis of lentivirus infected T cells by Flow cytometry. All sample were prepared after 5 days of virus infection.

The TF-CAR construct sequentially contains mlFVII, CD8 hinge and transmembrane domains, and the intracellular signaling domains of CD28, 4-1BB and CD3ζ (Figure 2A). We prepared the TF-CAR T cells through infecting human primary T cells with lentiviruses containing the encoding sequence of the TF-CAR.

On 5 days after infection, expression of the CAR in T cells was evaluated by western blot using an anti-CD3ζ antibody, which is able to recognize both the endogenous CD3ζ and the TF-CAR containing a CD3ζ domain. As expected, the endogenous CD3ζ chains were detected in all T cells, whereas the TF-CAR was only detected in TF-CAR-transduced T cells (Figure 2B).

To further examine TF-CAR expression on the surface of T cells, T cells were stained with a polyclonal goat IgG anti-mFVII primary antibody and a PE-conjugated mouse anti-goat secondary antibody. Then, the stained cells were detected by flow cytometry and fluorescence microscopy. According to the FACS analysis, the positive rate of TF-CAR was 57.0% Figure 2C). TF-CAR expression on the T cell membrane was also detected by fluorescence microscopy (Figure 2D).

### Cytokine release of TF-CAR T cells *in vitro*

**Figure 3:**
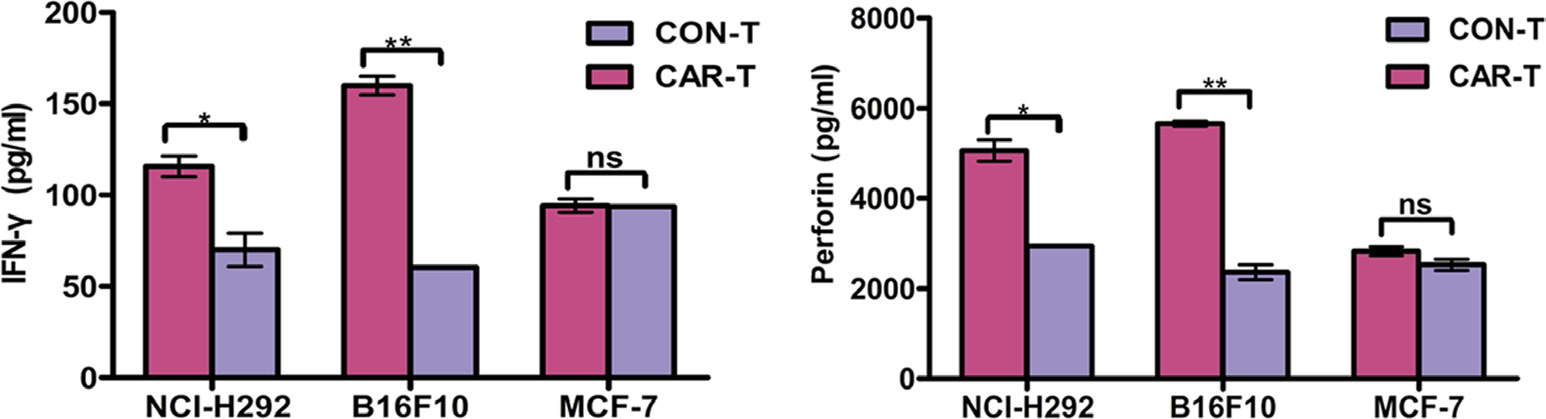
Cytokine release of TF-CAR T cells In total, 1 × 10^4^ TF-CAR T cells were co-cultured with 1 × 10^4^ tumor cells in 100 μl medium per well in round bottom 96-well-plate in triplicate. After 24 h, the levels of IFN-γ and perforin were evaluated by ELISA. *p < 0.05; **p < 0.01; ns, not significant.

To investigate whether TF-CAR T cells could specifically recognize target cells and acquire effector cell functions, a cytokine release assay was performed. As shown in Figure 3, compared with control T cells (CON-T), the TF-CAR T cells (CAR-T) co-cultured with NCI-H292 or B16F10 cells, but not with MCF-7 cells, released significantly increased amounts of INF-γ and perforin. These results indicated that the TF-CAR T cells could specifically recognize TF-positive target cells and elicit specific effector cell functions.

### Cytotoxicity of TF-CAR T cells *in vitro*

**Figure 4:**
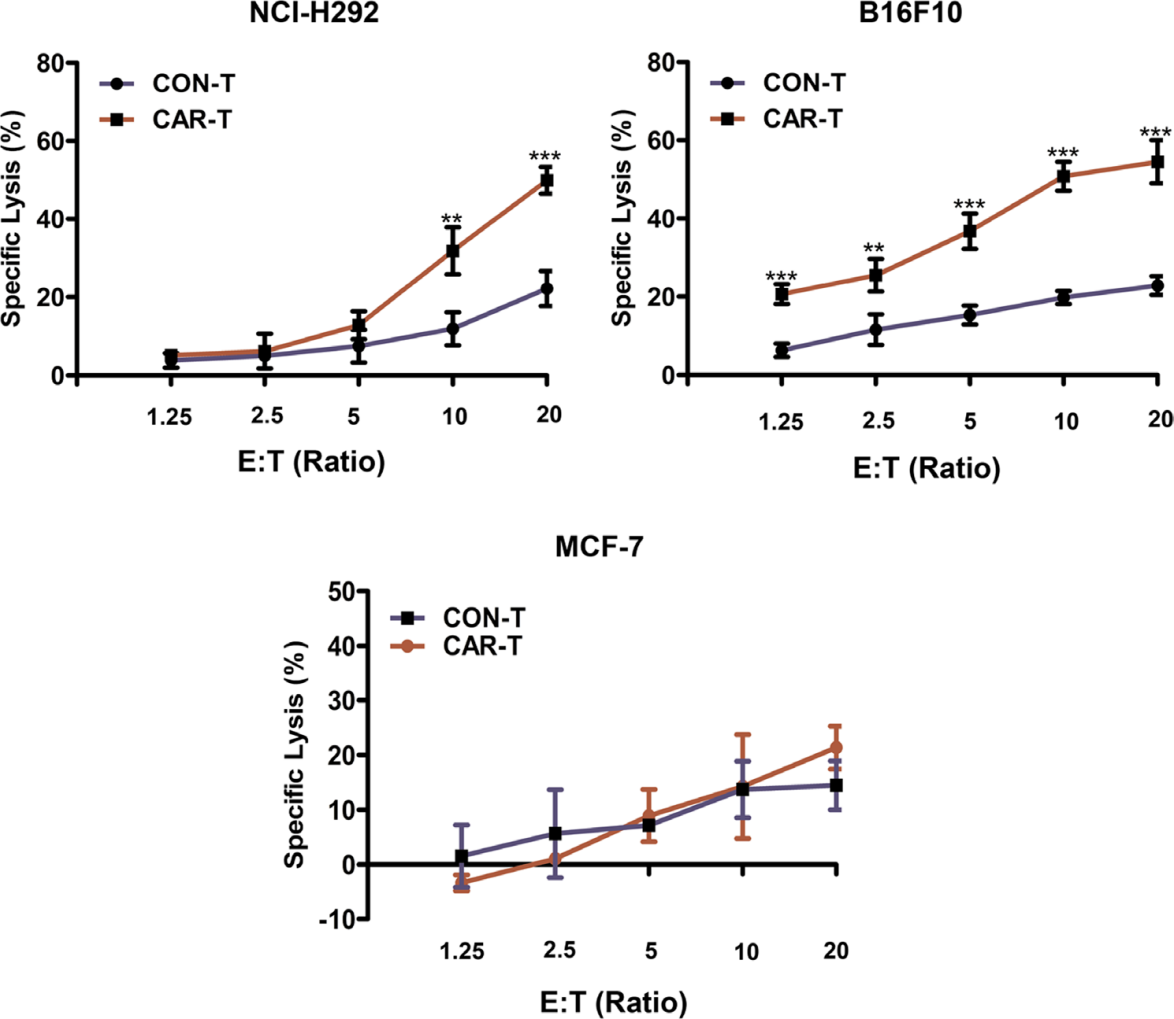
Cytotoxicity of the TF-CAR T cells against TF-positive cancer cells 5 × 10^3^ cancer cells were co-cultured with effector cells at the indicated effector : target (E:T) ratios in 100 μl per well in a 96-well-plate in triplicate. After 4 h, the released levels of lactate dehydrogenase (LDH) enzyme were evaluated. *p < 0.05; **p < 0.01; ***p <0.001; ns, not significant.

To determine whether TF-CAR T cells could specifically recognize and kill TF-positive cancer cells, LDH release assays were performed. The results indicated that, compared with CON-T cells, TF-CAR-T cells showed stronger killing activity against NCI-H292 and B16F10 cells. However, the cytotoxicity difference between the two T cell types against MCF-7 cells was not significant. Additionally, the cytotoxicity of TF-CAR T cells against TF-positive tumor cells was positively correlated with the effector:target ratios (Figure 4). These results further demonstrated that TF-CAR T cells could specifically recognize and kill TF-positive cancer cells.

### Growth suppression of established TF-positive NSCLC xenografts by TF-CAR T cells

To examine the therapeutic efficacy of TF-CAR T cells against TF-positive tumors, we established a subcutaneous xenograft model in NOG mice using the human NSCLC line NCI-H292 containing the gene encoding luciferase (NCI-H292-luc). First, we treated the mice with the TF-CAR T cells by i.v. injection once a week for three weeks. However, the therapeutic efficacy was not obvious at the end of the treatment (Supplementary Figure 1). One possible reason for this lack of therapeutic efficacy is that it was difficult for the TF-CAR T cells to traffic into the tumors [2].

**Figure 5:**
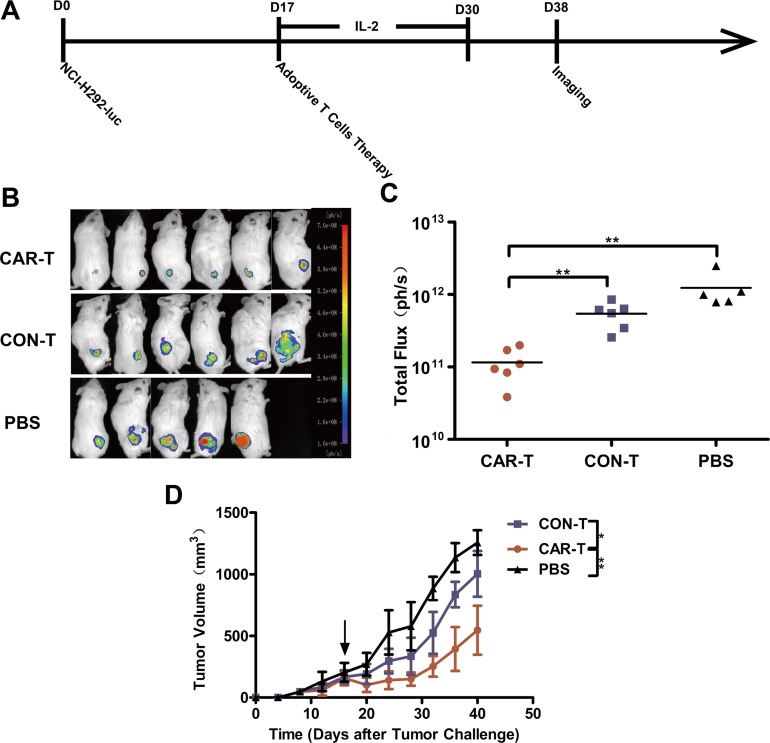
Growth suppression of established s.c. xenograft by TF-CAR T cells (**A**) Schematic diagram showing the treatment program of the mice. (**B**) Luminescence images showing the tumor size after adoptive cell therapy. (**C**) Quantitative results of the tumor luminescence intensity shown in (B). (**D**) The tumor growth curves during the experiment. Arrows indicate the time of T cells infusion.

To overcome this obstacle, we treated the mice with the TF-CAR T cells by intratumoral injection. The treatment program is shown in Figure 5A. To monitor tumor growth, we measured the tumor dimensions using calipers. On day 39, tumor sizes were also measured by *in vivo* imaging. As shown in Figure 5B and 5C, treatment with TF-CAR-T cells significantly suppressed tumor growth compared with the CON-T group and PBS group. The values of the tumor volume were concordant with those of the *in vivo* imaging. These data indicated that intratumoral injection of TF-CAR T cells resulted in significant inhibition of the growth of TF-positive NSCLC xenografts *in vivo*.

### Suppression of TF-positive NSCLC cell metastasis by TF-CAR T cells

**Figure 6:**
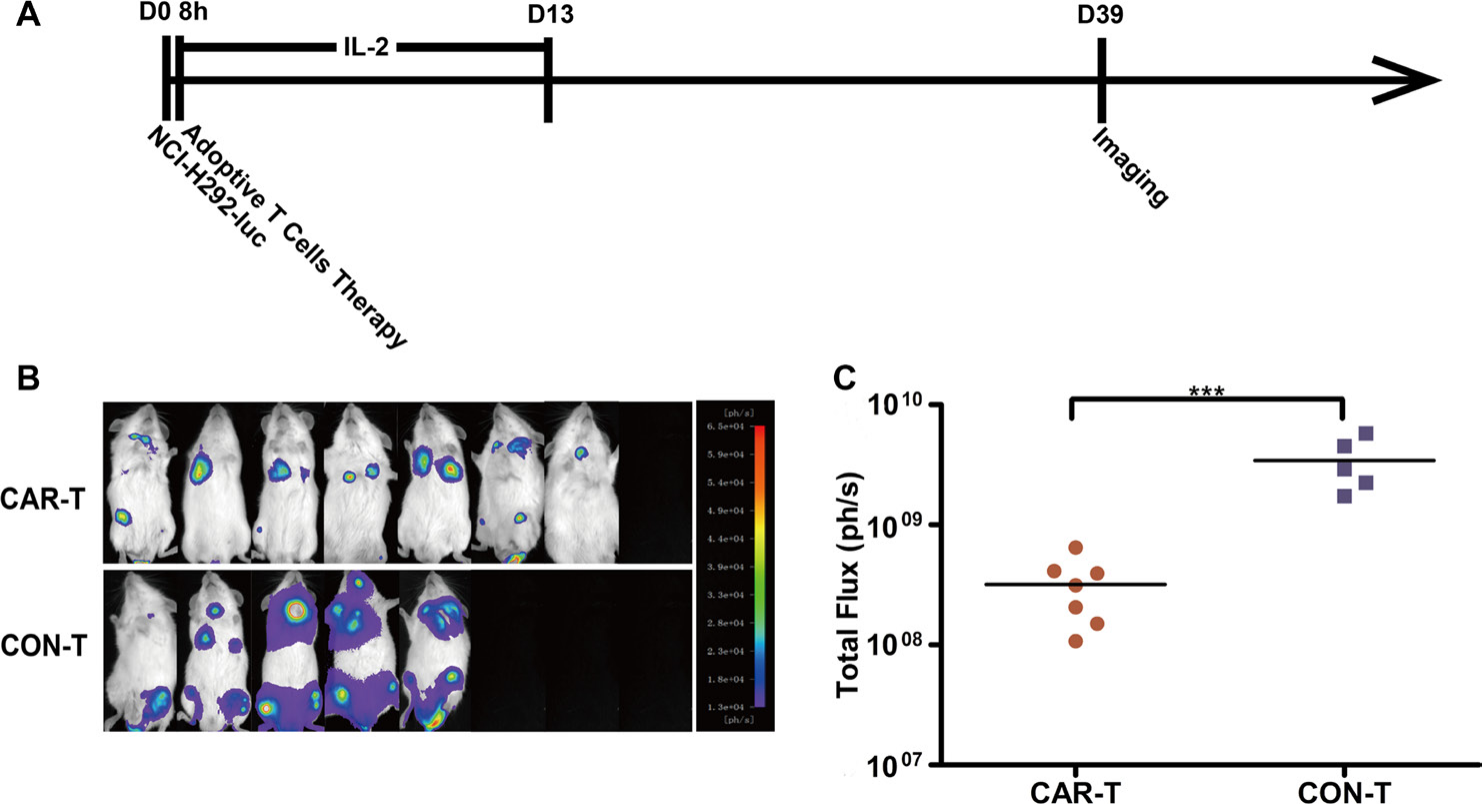
Metastasis suppression of TF-positive cancer cells by TF-CAR T cells (**A**) Schematic diagram showing the treatment program of the mice. (**B**) Luminescence images showing the metastatic tumors in the mice after adoptive cell therapy. (**C**) Quantitative results of the luminescence intensity of pulmonary metastatic tumors shown in (**B**). *n* = 8. *p < 0.05; ***p < 0.001.

We next evaluated the ability of TF-CAR-T cells to suppress TF-positive cancer cell metastasis *in vivo*. We established a pulmonary metastasis model of human NSCLC NCI-H292-luc cells in NOG mice by i.v. injection. The treatment program is shown in Figure 6A. Metastatic NCI-H292-luc cells were detected in the lung and bone of the mice treated with CON-T cells. In contrast, treatment with TF-CAR T cells significantly inhibited NCI-H292-luc cell metastasis, especially metastasis to bone; in the mice treated with TF-CAR T cells, only a few metastatic tumors were detected in the bone of the rear legs (Figure 6B). The statistical analysis of the luminescence intensity of pulmonary metastatic tumors showed that TF-CAR T cells significantly inhibited the pulmonary metastasis of NCI-H292-luc cells compared with CON-T cells (*P* < 0.001) (Figure 6C).

### Persistence of T cells in tumors

**Figure 7:**
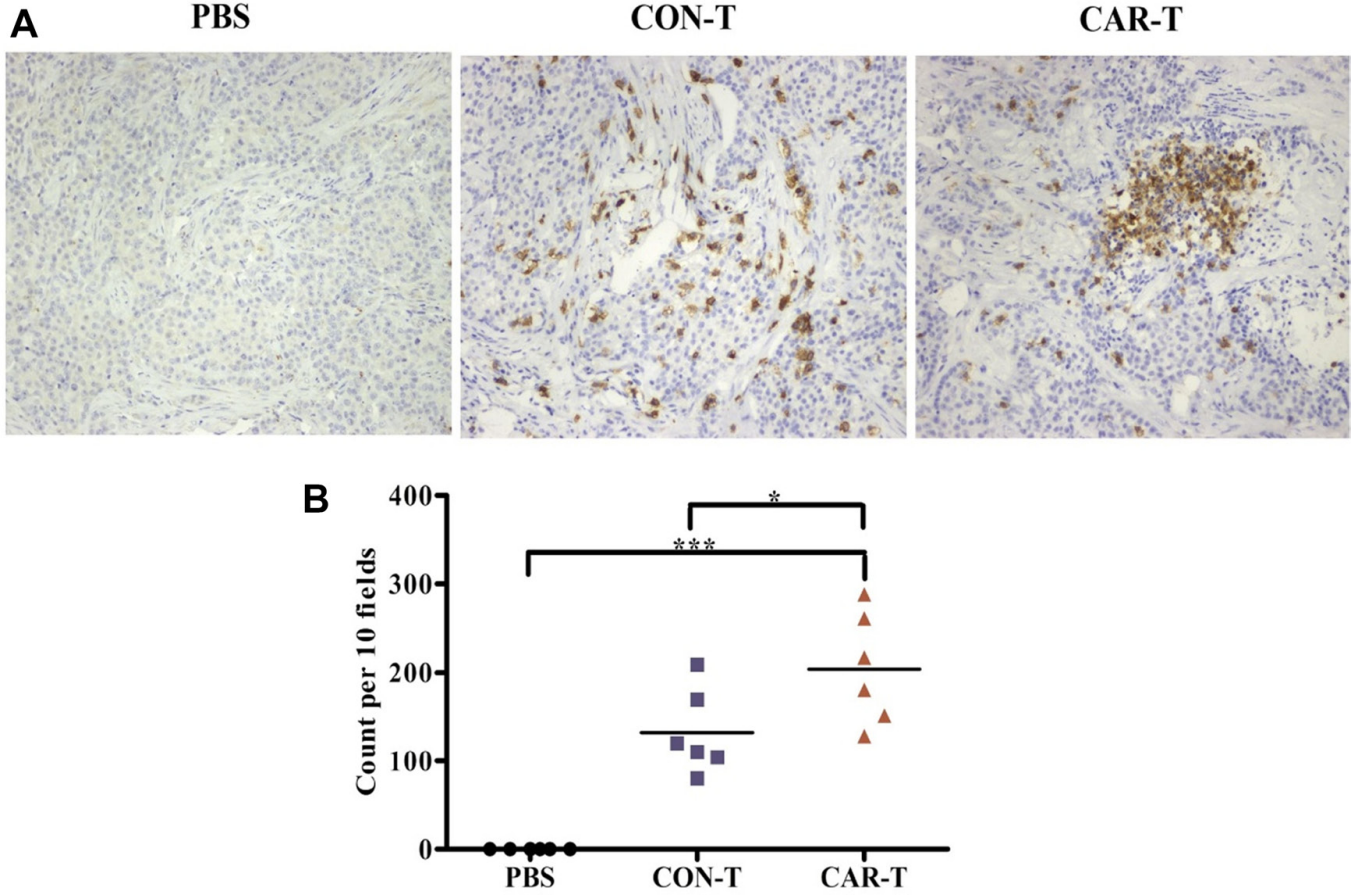
Persistence analysis of T cells *in vivo* (**A**) Immunohistochemical analysis of human CD3^+^ T cells in established s.c. xenografts. The images were obtained under × 200 magnification. (**B**) The corresponding quantitative analysis results of human CD3^+^ T cells shown in (A). *p < 0.05; ***p < 0.001; ns, not significant.

We next investigated the existence of T cells in tumor sites. For the mice treated by i.v. injection, few human CD3^+^ T cells were detected in either the CAR-T group or CON-T group (data not shown). In contrast, for the mice treated by intratumoral injection, human CD3^+^ T cells were detected in the tumor sites of the CAR-T group and CON-T group (Figure 7A). Furthermore, the number of CD3^+^ T cells in tumors of mice in the CAR-T group was higher than that in tumors of mice in the CON-T group (Figure 7B). These results suggested that tumor regression was associated with the existence of TF-CAR T cells in tumors.

### Safety of TF-CAR T cells *in vivo*

**Figure 8:**
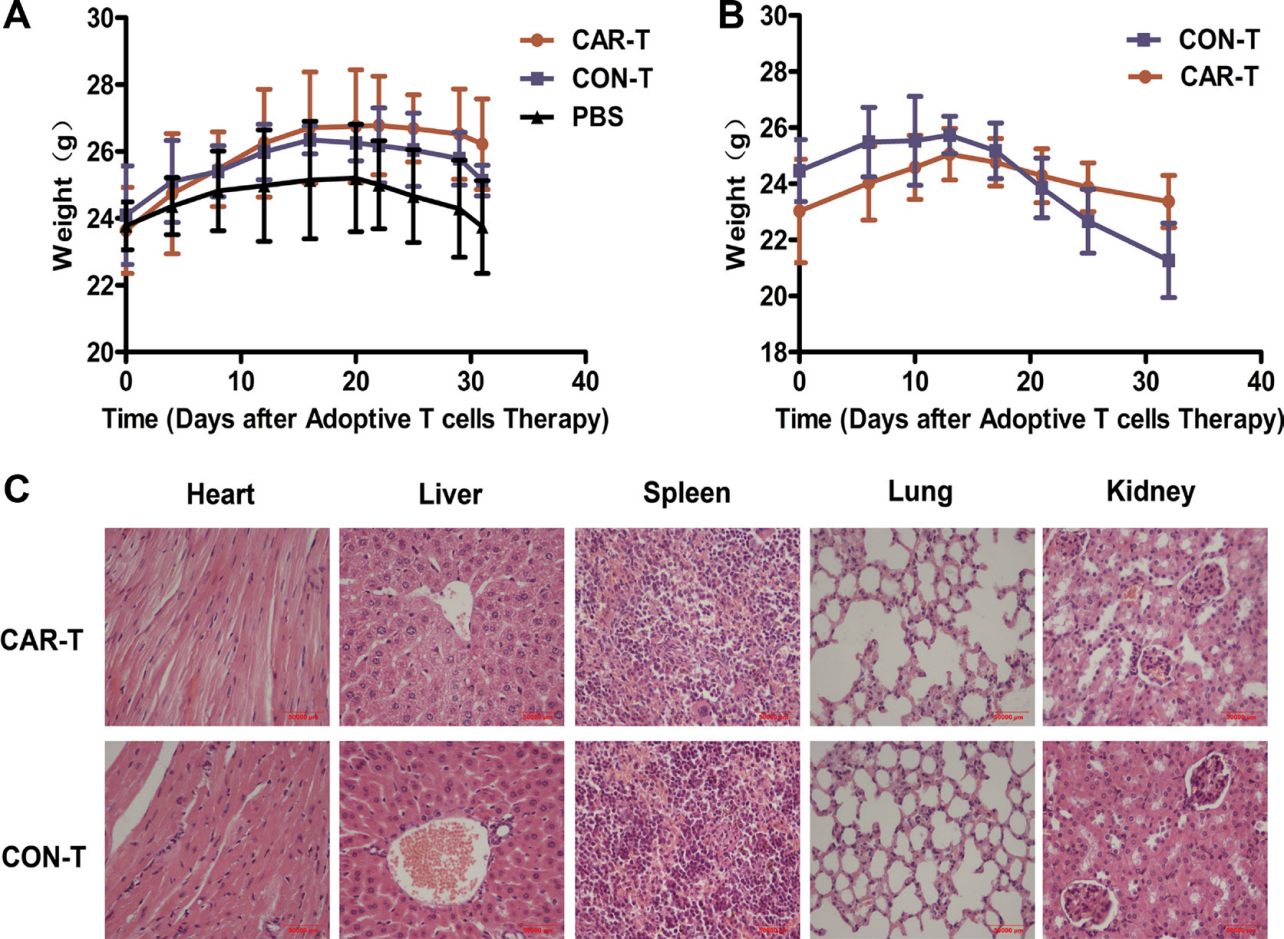
Body weight and pathological analysis of the mice received adoptive cell therapy (**A**) The body weight shift curve of the mice with s.c. xenografts during treatment. (**B**) The body weight shift curve of the mice with metastatic tumors during treatment. (**C**) Pathological analysis of important organs of the mice by Hematoxylin and eosin (HE) staining.

The body weight of the mice was monitored as a systemic safety indicator of the TF-CAR T cells *in vivo*. At the end of the tumor growth inhibition experiment, TF-CAR T cells was less than that of the mice treated with PBS and CON-T cells in both s.c. xenograft and lung metastasis experiments (Figure 8A, 8B). These results demonstrated that the TF-CAR T cells improved the quality of life of the mice.

On-target off-tumor toxicity is the main limitation of the application of CAR T cells in clinic. CAR T cells attack normal organs because of the tumor-associated antigen expression in these organs, causing serious side effects [30, 31] and even death in clinical trials [32]. To further evaluate the safety of TF-CAR T cells *in vivo*, we performed pathological inspections of important organs of the experimental mice that received adoptive cell therapy by i.v. injection; no obvious pathological changes were found in these organs. Representative histology of organ sections stained with HE are shown in Figure 8C. These results further demonstrated that the TF-CAR-T cells did not cause obvious side effects *in vivo*.

## DISCUSSION

TF overexpression is observed in clinical samples of numerous types of human cancers [10]. However, previous studies have featured conflicting reports regarding TF expression in lung cancer and melanoma. In this study, we demonstrated that TF is overexpressed in squamous cell carcinoma, adenocarcinoma and some other types of NSCLC; however, TF expression in small cell carcinoma is significantly lower than that in normal lung tissue (Table 1). The differential expression of TF in different types of tumors indicates that individual characteristics of each tumor are important factors that should be considered when using TF-targeted therapeutics, including TF-CAR T cells, to treat patients with lung cancer or melanoma.

Possible on-target off-tumor toxicities resulting from the expression of tumor-associated antigens on normal tissues can lead to killing of nontumor cells. If the high doses of CAR T cells that these tissues are exposed to with the initial cell injection result in killing cells in the heart, lung, or liver, then rapid death will occur [32]. Therefore, preclinical toxicity studies in animal models are important for the clinical application of CAR T therapies. However, common xenograft models using immunodeficient mice have thus far failed to efficiently support preclinical toxicity evaluations because of the antigen/receptor differences between humans and mice. In this study, we chose mlFVII as the target-binding domain of the TF-CAR. Because mFVII possesses equivalent affinity to mTF and hTF [23, 24], the TF-CAR T cells could attack both hTF-expressing tumor cells and mTF-expressing normal tissues in our mouse models; therefore, this system can stimulate the behavior of TF-CAR T cells with hlFVII as the target vehicle in the human body to a certain extent. Our data showed that after 3 infusions of TF-CAR T cells, no obvious pathological changes were detected in the important organs of the mice, such as the heart, liver, spleen, lung and kidney (Figure 8C). However, because of the differences in TF expression between humans and mice, additional in-depth studies are needed to confirm the safety of TF-CAR T cells before their application in patients.

For the treatment of s.c. xenografts in our mouse model, TF-CAR T cells were first administered systemically to the mice; however, the treatment only displayed limited therapeutic activity (Supplementary Figure 1). Then, we treated the mice bearing s.c. xenografts with TF-CAR T cells by intratumoral injection, and the therapeutic efficacy improved significantly (Figure 5); therefore, the limited efficacy of systemic administration was very likely due to the poor capacity of infused T cells to reach the tumor site. This result is in line with previous studies in animal models and clinical trials [33–35]. The lack of expression of chemokine receptors and the enzyme heparanase, which degrades heparin sulfate proteoglycans (the main components of the extracellular matrix), in *in vitro*-cultured T lymphocytes may be the main reason for its poor ability to penetrate into tumors [36, 37]. Therefore, the development of TF-CAR T cells engineered to express heparanase or chemokine receptors is one way to improve the therapeutic efficacy of TF-CAR T against TF-positive solid tumors.

The expression level of TF in metastatic cells may be up to a 1000-fold higher than that in nonmetastatic cells, suggesting a direct role for TF in tumor metastasis [38, 39]. In lung cancer, upregulation of TF correlates with tumor metastasis [40], and elevated circulating levels of tissue factor-positive microvesicles are associated with distant metastasis [41]. The NCI-H292 cell line used in this study was derived from a lymph node metastasis of a pulmonary mucoepidermoid carcinoma. Our data showed that TF-CAR T cells significantly inhibited NCI-H292 cell metastasis, especially metastasis to bone (Figure 6); therefore, TF-CAR T cells may be an effective approach to preventing metastasis of TF-positive cancers.

We constructed a new third-generation CAR targeting TF. Our data revealed that TF might be a rational target of NSCLC and melanoma. The TF-CAR T cells showed strong specific cytotoxicity against TF-positive cancer cells *in vitro* and effective growth and metastasis inhibition in a TF-positive cancer model *in vivo*. These results support future clinical testing of this therapeutic approach in patients with TF-positive malignancies.

## MATERIALS AND METHODS

### Human specimens and immunohistochemistry

The tissue microarrays of human samples were purchased from Shanxi ChaoYing Biotechnology Co., Ltd. Immunohistochemistry was performed according to the avidin biotinylated-HRP complex (ABC) method using a standard ABC kit (ZSGB-BIO, Beijing, China). Slides of the tissue microarrays were incubated with a polyclonal rabbit anti-TF antibody (1:200 dilution) (Bioword Technology, Inc.) overnight at 4°C. After the slides were washed, they were incubated with horseradish peroxidase-conjugated secondary antibody. Immunoreactivity was detected using the DAB staining system (ZSGB-BIO, Beijing, China).

Immunolabeling was scored separately for 2 variables: the number of TF-positive cells and the staining intensity. Scoring for the number of positive tumor cells was defined as follows: 1, < 25% positive cells; 2, 25–50% positive cells; 3, > 50–75% positive cells; 4, > 75% positive cells. Intensity scoring was defined as follows: 0, no staining; 1, light staining; 2, moderate staining; 3, intense staining. The slides were independently interpreted by 2 readers without knowledge of the clinical data. In the cases with a discrepancy between duplicated cores, the average score from the 2 tissue cores was taken as the final score. The level of relevant protein staining was evaluated using an immunoreactive score (IRS), which is calculated by multiplying the staining intensity scores and the percentage of positive cells.

### Cell lines

The human NSCLC cell line NCI-H292, mouse melanoma cell line B16F10 and human breast cancer cell line MCF-7 were purchased from the Institute of Biochemistry and Cell Biology, Chinese Academy of Sciences (Shanghai, China). NCI-H292 and B16F10 cells were cultured in RPMI-1640 medium. MCF-7 cells were cultured in DMEM medium. Both media were supplemented with 10% fetal bovine serum (FBS) and 1% penicillin/streptomycin. All media, FBS and penicillin/streptomycin were purchased from Gibco (Grand Island, NY, USA). The NCI-H292-luc cells were generated by transduction of NCI-H292 with a recombinant lentivirus encoding luciferase of *Photinus pyralis*.

### Construction of TF-CAR and lentivirus preparation

The TF-CAR construct (LV5-mlFVII-CD8-28BBZ) is composed of mlFVII, CD8 (138-208 aa), CD28 (180-220 aa), 4-1BB (214-255 aa) and CD3ζ (52-164 aa) domains. The encoding sequence of the CAR was inserted into the lentiviral vector LV5 (Gene Pharma, Shanghai, China) with an EF1-alpha promoter. To produce lentiviral particles, the plasmid LV5-mlFVII-CD8-28BBZ was transduced into 293T cells with pMD2.G and psPAX2. 48 hours after transfection, viral supernant was harvested and spun at 4°C, 2000 rpm for 7 min. The viral supernant then was filtered through 45 μm filter. The filtered supernant was spun at 4°C, 70000 g for 2 hours. Supernant was discarded. Pellet was resuspended in cold PBS with volume of 1/100 initial viral supernant. The virus was aliquoted, flash-frozen in liquid nitrogen and stored at −80°C. Viral titer was determined using a Lenti-X p24 Rapid Titer Kit (Clontech, CA, USA).

### Generation and transduction of T cells

Peripheral blood mononuclear cells (PBMCs) were separated from peripheral blood of healthy donors using Human Lymphocyte Separation tubes (Dakewe Biotech Company Ltd., Shenzhen, China). PBMCs were cultured in AIM-V medium (Gibco, Grand Island, NY, USA) supplemented with 5% human AB serum (Innovative Research, Inc), 200 IU/ml recombinant human IL-2 (rhIL-2) (PeproTech, Rocky Hill, NJ). For lentivirus infection, the PBMCs were activated using Dynabeads Human T-Activator CD3/CD28 (Life Technologies, #111310) at a 1:3 cell:bead ratio in 48-well plate (0.25 million cells/well). After 24 h, lentivirus (MOI = 5) and polybrene at a final concentration of 8 μg/ml were added into the well. The plate was centrifuged at 32°C, 1500 g for 2 hours. The cells were check every day. On day 5 post activation, the beads were withdrawn, the CAR expression was detected.

### Western blot analysis

Western blot analysis was performed to measure the expression of TF-CAR in T cells. 5 days after gene transduction, total proteins were extracted from the T cells and were separated on a 10% SDS polyacrylamide gel. The proteins were then transferred to nitrocellulose membranes and incubated overnight at 4°C with a primary rabbit anti-human CD3ζ antibody (Abcam, Beverly, MA, USA). Next, the membranes were washed and incubated with a goat anti-rabbit IgG (H+L) secondary antibody (VICMED Company, Xuzhou, Jiangsu, China) at 37°C for 1 hour. Images were captured and analyzed using the Tanon 5200 Chemiluminescent imaging system (Tanon, Shanghai, China).

### Flow cytometry

In total, 1 × 10^6^ cells were suspended in 0.1 ml fluorescence-activated cell sorter (FACS) buffer (2% FBS in PBS) and stained with fluorescent-labeled antibodies for 30 minutes at room temperature. The cells were then washed, suspended and evaluated with a FACS machine (FACSCanto II, Becton-Dickinson, USA). All data were analyzed by FlowJo software (Tree Star, USA). The following antigens and antibodies were used for this analysis: human TF, mouse anti-human TF primary antibody (R&D Systems, Minnesota, USA) and FITC-conjugated anti-mouse IgG1 secondary antibody (eBioscience, San Diego, CA); mouse TF, PE-conjugated goat anti-mouse TF antibody (R&D Systems, Minnesota, USA); TF-CAR, polyclonal goat anti-mouse coagulation factor VII primary antibody (R&D Systems, Minnesota, USA) and PE-conjugated rabbit anti-goat IgG secondary antibody (Santa Cruz, CA, USA). Matched isotype control antibody was used in all analyses.

### Immunofluorescence staining

Cell-surface expression of TF-CAR was also detected by immunofluorescence staining. 5 days after transduction, T cells were stained as previously described and analyzed by flow cytometry. Cell nuclei were stained with Hoechst Trihydrochloride (Thermo Fisher Corp., San Jose, CA, USA). Then, the cells were fixed on slides coated with 0.1 mg/ml polylysine (Sigma-Aldrich, St. Louis, MO, USA). The images were taken using a fluorescence microscope (DS-Ri1, Nikon, Japan).

### Cytokine release assay

In total, 1 × 10^4^ target cells (NCI-H292, B16F10 or MCF-7) were mixed with TF-CAR T cells or control lentivirus-infected T cells (CON-T) at a 1:1 effector cell:target cell ratio. Then, the mixed cells were added to the round bottom of a 96-well plate with 200 μl medium. Twenty-four hours later, supernatants were harvested. IFN-γ and perforin were detected using ELISA kits (Dakewe Biotech Company Ltd., Shenzhen, China) according to the manufacturer's instructions.

### Cytotoxicity assay

In total, 5 × 10^3^ target cells and effector cells at ratios of 1:1.25, 1:25, 1:5, 1:10 and 1:20 were added to the round bottom of 96-well plates with a final volume of 100 μl. After the cells were spun down, they were co-cultured for 4 hours in a 37°C incubator. The released LDH was detected using a CytoTox 96^®^ Non-Radioactive Cytotoxicity Assay Kit (Promega Corp., Madison, WI, USA). The specific lysis activity was calculated by the following formula: % Cytotoxicity = (Experiment−Effector Spontaneous−Target Spontaneous)/(Target Maximum−Target Spontaneous) ×100%.

### Mice and *in vivo* experiments

*In vivo* experiments involved 6–8 week-old female NOG (NOD/Shi-scid, IL-2Rγnull) mice (Vital River Laboratory Animal Technology Co., Ltd., Beijing, China), which were housed in the specific pathogen-free animal facility of the Experimental Animal Center, Xuzhou Medical University, China. All experimental animal procedures were performed in compliance with the institutional ethical requirements and approved by the Committee of Xuzhou Medical University for the Use and Care of Animals. All animal experimental protocols were approved and reviewed by the Institutional Animal Care and Use Committee of the Jiangsu Provincial Academy of Chinese Medicine (SCXK2012–0005).

### Human NSCLC s.c. xenograft mouse model

In total, 3 × 10^6^ NCI-H292-luc cells were injected s.c. on the right flank of NOG mice on day 0. When the tumors had grown to 150–200 mm^3^ (day 17), the mice were divided into three groups (*n* = 6) and received 1 × 10^7^ CAR-T cells, 1 × 10^7^ CON-T cells or PBS by i.v. or intratumor injection. From the day of T cell infusion, all mice were administered 2000 IU IL-2 daily by intraperitoneal (i.p.) injection for two weeks. The tumor dimensions were measured using calipers. Tumor volumes were calculated using the formula V = 1/2 × (length×width^2^), where the length is the greatest longitudinal diameter and the width is the greatest transverse diameter. On day 38, tumor size was monitored by bioluminescent imaging (BLI). During the treatment, the body weights of the mice were recorded. At the experimental endpoint, when the tumor volumes reached approximately 2,000 mm^3^ in the control groups, the mice were euthanized.

The harvested organs and tumors were fixed in 10% neutral buffered formalin, embedded in paraffin, and cut into 3–5 μm sections [42]. Human CD3^+^ T cells in tumors were detected by IHC as described above using a rabbit anti-human CD3 antibody (Abcam, Beverly, MA, USA) at a 1:200 dilution. For the quantification of human CD3^+^ T cells in the tumors, T cells were counted in 10 randomly selected intratumoral fields of each slide under × 200 magnification. Histological changes in the organs, including heart, liver, spleen, lung and kidney, were examined by hematoxylin and eosin (HE) staining. Imaging was performed using a fluorescence microscope (DS-Ri1, Nikon, Japan).

### Human NSCLC lung metastasis mouse model

For the lung metastasis model, the mice were divided into two groups (*n* = 8). On day 0, each mouse was injected with 1 × 10^6^ NCI-H292-luc cells intravenously (i.v.) through the tail vein. Eight hours later, each mouse in the two groups received 1 × 10^7^ CAR-T or CON-T cells by i.v. injection. In total, 2000 IU IL-2 was administered to each mouse by i.p. injection daily for two weeks from the day of T cell infusion. On day 39, metastasis tumors were monitored by BLI. During the treatment, body weight were recorded.

### Statistical analysis

The data were analyzed using SPSS (version 16.0, SPSS Inc., Chicago, IL, USA) and GraphPad software (GraphPad, San Diego, CA, USA). Differences in IRS for TF staining in primary tumors and their paired normal tissues were assessed by independent-sample *t*-test. Unless otherwise noted, the data are summarized as the mean ± standard deviation (SD). A comparison between two groups was performed by independent-sample *t*-test, while multiple samples were compared by one-way ANOVA, with α = 0.05 as the significance level for the test. The results were considered statistically significant at *P* < 0.05. No valuable samples were excluded from the analyses. Animals were excluded only in the event of their death after tumor implantation but before T cell infusion.

## SUPPLEMENTARY FIGURE


