# Supplementary material for: Chimeric antigen receptor-modified T Cells inhibit the growth and metastases of established tissue factor-positive tumors in NOG mice
Source: Oncotarget. 2016 Dec 30;8(6):9488–99. doi: 10.18632/oncotarget.14367 (PMC5354747; doi:10.18632/oncotarget.14367)
Supplement: Supplementary file 1 [file oncotarget-08-9488-s001.pdf]

Chimeric antigen receptor-modified T Cells inhibit the growth and metastases of established tissue factor-positive tumors in NOG mice

Supplementary Materials

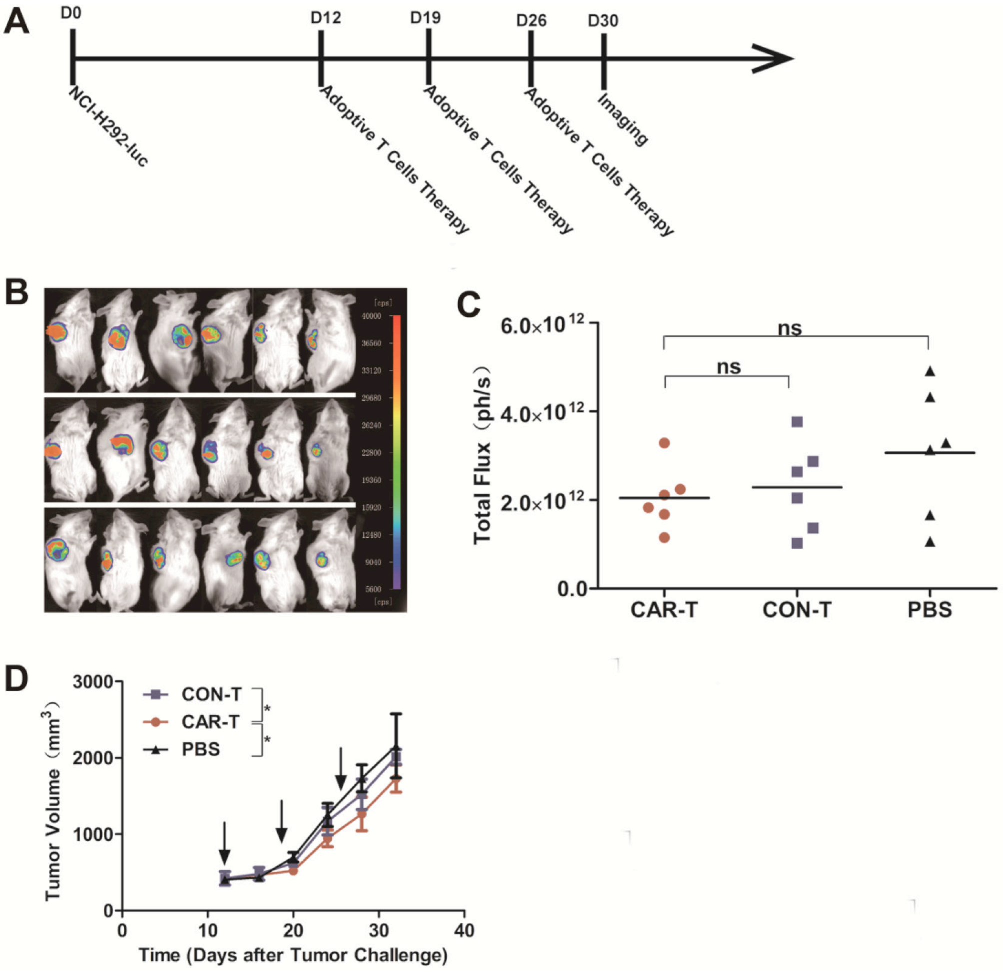

Supplementary Figure 1: Growth suppression of established s.c. xenograft by i.v. injected TF-CAR T cells. (**A**) Schematic diagram showing the treatment program of the mice. (**B**) Luminescence images showing the tumor size after adoptive cell therapy. (**C**) Quantitative results of the tumor luminescence intensity shown in (B). (**D**) The tumor growth curves during the experiment. Arrows indicate the time of T cells infusion.
